# Understanding the Drivers of Strike Actions Among Nurses in Ghana: A Qualitative Study

**DOI:** 10.1002/nop2.70475

**Published:** 2026-03-04

**Authors:** Ba‐Etilayoo Atinga, Christiana Asospae Ayamga, Vincentia Sarfo‐Brobbey, Albert Henyo, Tulukuu Perekuu, Rosemary Braimah

**Affiliations:** ^1^ Department of Nursing, School of Sciences University of Energy and Natural Resources Sunyani Ghana; ^2^ Nursing, Holy Family Nursing and Midwifery Training College Berekum Ghana

**Keywords:** Ghana, healthcare workforce, industrial action, nurse strike, nursing profession, qualitative research

## Abstract

**Aim:**

This study aimed to explore the rationale behind nurses' decisions to strike, focusing on the lived experiences, motivations, and institutional conditions that prompt such industrial actions.

**Design:**

A qualitative exploratory study.

**Methods:**

A semi‐structured interview was conducted with 20 professional nurses working with a district, regional, or teaching hospital who have ever engaged in strike. Participants were selected from public hospitals across 5 regions in Ghana based on their consent to participate in the study across Ghana between March 2025 and June 2025. The data were analysed using Braun and Clarke's method.

**Results:**

Data analysis led to five themes and fifteen subthemes. These include poor remuneration, lack of adequate logistics, perceived government neglect, professional identity struggles, and emotional exhaustion.

**Conclusion:**

The findings highlight the urgent need for policy interventions that address systemic inequities and improve the professional welfare of nurses.

**Patient or Public Contribution:**

No patient or public contribution.

## 1 Introduction

Industrial actions by nurses have become a recurring and highly visible phenomenon within Ghana's public healthcare sector, frequently disrupting service delivery and exposing the structural fragilities of the national health system (Buabeng‐Baidoo and Olivier 2024). In Ghana, where public health services constitute the primary source of care for the majority of the population, nurses' strikes have far‐reaching consequences for patient outcomes, public trust, and health system resilience (Ampofo et al. 2022). Although industrial action is typically framed as a measure of last resort, recent years have witnessed an escalation in the frequency, duration, and intensity of nurses' strikes, suggesting unresolved systemic grievances that require closer scholarly interrogation (Essex et al. 2023).

Within the Ghanaian health system, professional nurses are defined as individuals who have completed accredited nursing education, are licensed by the Nursing and Midwifery Council of Ghana, and are legally authorised to provide professional nursing care across preventive, curative, and rehabilitative settings. These nurses constitute the largest cadre of the country's health workforce and serve as the backbone of frontline healthcare delivery in hospitals, clinics, and community settings (Nyande et al. 2024). Despite their central role, professional nurses in Ghana are frequently driven to industrial action due to persistent challenges, including poor remuneration, unsafe and resource‐constrained working environments, limited opportunities for career progression, lack of professional recognition, and repeated delays in the fulfilment of negotiated government commitments (Sony et al. 2025; Nanthagopan and Vivek 2021; Owusu 2021).

Labour relations scholarship suggests that sustained industrial unrest within essential services is rarely the product of isolated dissatisfaction; rather, it reflects deeper institutional dysfunctions, governance failures, and power asymmetries embedded within employment systems (Dobbins 2023). In Ghana, public sector wage negotiations and labour disputes are historically politicised, and nurses' strikes often symbolise broader tensions related to healthcare financing, inequitable human resource management, and the structural marginalisation of nursing within policy decision‐making spaces (Briskin 2012). These dynamics situate nurses' industrial actions not merely as economic protests but as expressions of systemic disempowerment within a constrained health system.

Beyond material conditions, nurses in Ghana shoulder a substantial emotional, ethical, and relational burden arising from chronic understaffing, moral distress, and the expectation to deliver quality care in the absence of adequate institutional support. Prolonged exposure to such conditions has been associated with moral injury, psychological exhaustion, and professional alienation—factors that may culminate in collective withdrawal from work as both a coping strategy and a form of resistance (Kayaalp et al. 2021). Understanding nurses' strikes therefore requires an analytical approach that moves beyond individual dissatisfaction to examine the interconnected personal, organisational, and policy‐level forces that shape nurses' lived realities.

Against this backdrop, this study explores the lived experiences and perspectives of professional nurses who have participated in industrial actions in Ghana. By situating nurses' strikes within their socio‐political, institutional, and professional contexts, the study seeks to illuminate the underlying drivers and systemic dynamics that sustain labour unrest in the Ghanaian healthcare sector. Such insights are critical for informing contextually responsive labour policies, strengthening workforce morale, and safeguarding the ethical foundations of care delivery within a resource‐constrained health system (Antwi et al. 2023; Aberese‐Ako 2016).

## 2 The Socio‐Ecological Model

This study is guided by the Socio‐Ecological Model (SEM), originally developed by Urie Bronfenbrenner and widely applied in health, labour, and organisational research to explain how individual behaviour is shaped by multiple, interacting layers of influence (Navarro and Tudge 2023). The model posits that human actions cannot be adequately understood in isolation but must be examined within nested systems, including individual, interpersonal, organisational, community, and policy environments.

The application of the Socio‐Ecological Model to nurses' industrial actions is supported by a growing body of literature demonstrating its utility in understanding workforce behaviour, occupational stress, and collective action in healthcare settings (Uchendu et al. 2020). Previous studies have employed the SEM to examine nurse burnout, job dissatisfaction, moral distress, and workforce attrition, highlighting how personal resilience is profoundly shaped by organisational cultures, leadership practices, labour policies, and broader political economies of health (Kayaalp et al. 2021; Aberese‐Ako 2016; Antwi et al. 2023). These studies underscore the relevance of an ecological approach for unpacking complex labour phenomena in low‐ and middle‐income country contexts.

Within this framework, nurses' strikes in Ghana are conceptualised not as isolated acts of defiance or professional irresponsibility, but as multi‐layered responses to entrenched systemic failures (Buabeng‐Baidoo and Olivier 2024). At the individual level, factors such as emotional exhaustion, moral injury, and professional identity strain influence nurses' willingness to engage in industrial action (Karakachian and Colbert 2019). At the interpersonal and organisational levels, strained nurse‐manager relationships, inadequate staffing, unsafe working conditions, and perceived institutional neglect exacerbate dissatisfaction. At the policy and structural levels, delayed negotiations, weak enforcement of labour agreements, inequitable remuneration structures, and limited political prioritisation of nursing concerns create conditions that normalise recurrent industrial unrest (Shiviti 2020).

By integrating these multiple levels of influence, the Socio‐Ecological Model provides a robust conceptual scaffold for organising data collection, analysis, and interpretation. It enables a nuanced understanding of nurses' industrial actions as rational, contextually grounded responses to intersecting individual, institutional, and systemic pressures. In doing so, the model aligns closely with the study's aim of advancing evidence‐based policy dialogue and fostering more equitable and sustainable labour relations within Ghana's healthcare sector.

## 3 Methodology

### 3.1 Participants and Sampling Procedure

A purposive sampling technique was used to recruit 20 registered professional nurses and midwives who had participated in at least one organised strike or industrial action. Participants were identified through professional networks, nursing associations, and referrals from hospital union representatives. This approach ensured the inclusion of information‐rich cases with direct experiential knowledge of the phenomenon under study.

The study purpose was achieved across the selected regions by ensuring proportional representation of participants from each ecological belt and healthcare facility level, thereby capturing diverse institutional experiences and policy exposures. Recruitment continued until data saturation was reached, defined as the point at which no new themes emerged from successive interviews.

### 3.2 Inclusion and Exclusion Criteria

Inclusion criteria comprised registered nurses or midwives licensed by the Nursing and Midwifery Council of Ghana, actively practicing in a public secondary‐ or tertiary‐level hospital, and with direct experience of participation in at least one industrial action. Participants were required to demonstrate willingness to voluntarily participate and to share their experiences.

Exclusion criteria included student nurses and interns, who typically lack formal involvement in union activities and strike decision‐making processes. Nurses employed in private healthcare facilities were excluded due to differences in governance, labour relations, and regulatory oversight. Individuals who declined consent, withdrew during the study, or could not be reached after repeated follow‐up attempts were also excluded.

### 3.3 Data Collection

Data were collected between March and June 2025 using semi‐structured interviews conducted face‐to‐face and via telephone, depending on participant availability and geographic location. Interviews lasted between 30 and 60 min and were conducted in English by the lead researcher in settings that ensured privacy and participant comfort.

Interviews began with general, open‐ended questions and were guided by participants' responses. A key opening question was: “What are the main factors or conditions that lead nurses like yourself or your colleagues to consider going on strike?” All interviews were audio‐recorded with participant consent using a Philips DVT4110 VoiceTracer recorder and transcribed verbatim.

Prior to each interview, the researcher reviewed the study information sheet and consent form with participants, explaining the study purpose, confidentiality measures, and the voluntary nature of participation, including the right to withdraw at any point without consequence.

### 3.4 Data Analysis

Data were analysed using thematic analysis, following Braun and Clarke's six‐phase framework (Braun and Clarke 2021). This approach was selected due to its flexibility and suitability for identifying patterned meanings across qualitative datasets. NVivo version 14 was used to manage, organise, and code the data.

The lead researcher conducted the initial coding after repeated reading and familiarisation with the transcripts. Codes were then grouped into categories and refined into themes through iterative comparison. To enhance analytical credibility, three qualitative research experts independently reviewed selected transcripts and emerging themes. Discrepancies were discussed and resolved through consensus, ensuring analytical transparency and coherence.

### 3.5 Ethical Considerations

Ethical approval was obtained from the Committee on Human Research, Publications and Ethics (CHRPE) of [The University of Energy and Natural Resources, Sunyani]. Formal permission was also obtained from the management of the selected hospitals prior to data collection.

Participants provided written informed consent and were assured of confidentiality, anonymity, and secure data handling. Identifiers were removed from transcripts, and audio files were stored on password‐protected devices accessible only to the research team.

### 3.6 Rigour and Trustworthiness

Several strategies were employed to ensure methodological rigour in accordance with Lincoln and Guba's criteria for trustworthiness. Credibility was enhanced through prolonged engagement with participants, memo writing during data collection and analysis, and member checking with selected participants to confirm transcript accuracy and theme interpretation. Dependability was ensured through detailed documentation of the research process and the use of an audit trail. Confirmability was strengthened by independent coding by a second researcher and regular peer debriefing sessions to challenge assumptions and minimise researcher bias. Transferability was supported through rich, contextualised descriptions of the study setting, participants, and findings.

## 4 Results

The study identified five major themes and fifteen subthemes (Table 2) related to the rationale behind nurses' decisions to strike, focusing on the lived experiences, motivations, and institutional conditions that prompt such industrial actions. These themes included: (1) poor remuneration and financial insecurity (2) lack of essential resources (3) government neglect and broken promises (4) struggle for professional recognition (5) emotional and psychological exhaustion. The qualitative findings, a literature review on the topic was conducted, and the data were analysed. As a result, the results and discussion were combined, with relevant quotes and supporting research used to substantiate the findings.

### 4.1 Demographic Characteristics of Participants

A total of 20 nurses from five regions across Ghana participated in this qualitative study exploring the drivers of strike actions. The sample comprised 12 females and 8 males, reflecting the gender distribution typically seen in the nursing profession. Participants spanned a wide age range, with 8 aged 21–30 years, 5 aged 31–40, another 5 aged 41–50, and 2 above 50 years. This age diversity offered generational perspectives on labor unrest within the profession. In terms of work experience, 5 participants had 1–5 years of service, 8 had 5–10 years, 4 had 11–20 years, and 3 had worked for over two decades. Such variation in tenure allowed for nuanced insights into how years of service shape nurses' attitudes toward industrial action.

Educationally, most participants held a diploma in nursing (*n* = 10), while 4 were certificate‐holding enrolled nurses, another 4 held bachelor's degrees, and 2 had postgraduate qualifications. These academic differences shed light on how educational levels influence career expectations and frustrations with limited advancement opportunities. Participants also occupied a range of professional ranks, including 4 senior enrolled nurses, 5 staff nurses, 5 senior staff nurses, and 3 nursing officers. Higher‐ranking roles were also represented by 1 senior nursing officer, 1 principal nursing officer, and 1 deputy director of nursing services. This rank‐based diversity enriched the study's understanding of how institutional hierarchy and recognition contribute to strike motivations (Table 1).

**TABLE 1:** Background characteristics of respondents.

Characteristics of nurse participants (*N* = 20)	Category	Frequency (*N*)
Sex	Male	8
	Female	12
Age	21–30	8
	31–40	5
	41–50	5
	Above 50	2
Years of experience	1–5	5
	5–10	8
	11–20	4
	21 and more	3
Education	Certificate (enrolled nurses)	4
	Diploma	10
	Bachelors	4
	Masters and above	2
Rank	Senior enrolled nurse	4
	Staff nurse	5
	Senior staff nurse	5
	Nursing officer	3
	Senior nursing officer	1
	Principal nursing officer	1
	Deputy director of nursing service	1

## 5 Results

The study identified five main themes and ten subthemes related to the rationale behind nurses' strike in Ghana. These themes were: (1) Poor Remuneration and Financial Insecurity, (2) Lack of Essential Resources, (3) Government Neglect and Broken Promises, (4) Struggle for Professional Recognition, and (5) Emotional and Psychological Exhaustion (Table 2). The qualitative findings were analysed and compared with existing literature on the topic. As a result, the findings and discussion were presented together, supported by relevant participant quotes and related research.

**TABLE 2:** Summary of emerged themes and subthemes.

S/N	Themes	Subthemes
1	Poor remuneration and financial insecurity	1.1. Salaries not commensurate with workload 1.2. Erosion of purchasing power through inflation 1.3. Delayed and inconsistent allowance payments
2	Lack of essential resources	2.1. Chronic shortages of drugs and consumables 2.2. Malfunctioning or obsolete equipment 2.3. Personal out‐of‐pocket purchasing of supplies
3	Government neglect and broken promises	3.1. Unfulfilled collective bargaining agreements 3.2. Communication gaps and perceived disregard 3.3. Politicisation of health‐sector negotiations
4	Struggle for professional recognition	4.1. Hierarchical marginalisation within the health team 4.2. Public misconceptions about nursing roles 4.3. Limited career advancement opportunities
5	Emotional and psychological exhaustion	5.1. Compassion fatigue and burnout 5.2. Moral distress over compromised care 5.3. Strike as a form of self‐preservation

### 5.1 Poor Remuneration and Financial Insecurity

#### 5.1.1 Salaries Not Commensurate With Workload

Nurses consistently expressed frustration about the mismatch between their heavy workloads and inadequate salaries. Many reported managing multiple wards and working night shifts with minimal compensation, often insufficient to meet basic living expenses. This is seen in a participant's narration.

We have a lot to do when we report to work. Sometimes I manage two wards on the night shift especially in wards that have both surgical and medical units together, yet my salary can't even cover a month's rent in the cities. —P7

Another participant puts it

We take a lot of risk in the ward—we are exposed to infections yet with limited PPE to protect you. When I consider all this risk with the meagre pay received at the end of the month, it feels like an insult. —P3

#### 5.1.2 Erosion of Purchasing Power Through Inflation

Participants described how economic instability and inflation severely eroded the real value of their salaries, making it difficult to afford essentials despite working full‐time in public health facilities. This is seen in the narration:

Until the needful is done we continue to suffer. The exchange rate usually dwarfs our salaries. The cedi‐dollar rate worsens our plight. In fact, every payday I recalculate the exchange rate and realize my value has dropped significantly again. —P11

And reflected in

We don't have a different market. It is the same market that everyone buys from—the rich and the poor, the politician and the voter. Prices of basic food items needed to keep soul and body together are rising faster than our salary increments. —P18

#### 5.1.3 Delayed and Inconsistent Allowance Payments

A recurring concern was the chronic delay or complete absence of allowances promised to nurses, especially in rural areas or for night duties, which further compounded financial stress. A participant puts it

The rural allowance promised us seems to be non‐existent now. You miss your way by accepting posting to the rural setting. The last time I had my rural posting allowance, it was eight months late; by then I'd borrowed to survive as it had already lost its significance. —P4

It also stressed by participant 15

Some time ago we had night duty allowance and beverages given as incentives for observing the night duties. At first, it was faced with much delay that even sometimes we turned to forget we were entitled to it. —P15

### 5.2 Lack of Essential Resources

#### 5.2.1 Chronic Shortages of Drugs and Consumables

Nurses repeatedly described having to improvise basic care because wards lacked gloves, syringes, and even first‐line medications. These shortages undermined professional confidence and strained nurse–patient relationships, as families were frequently asked to purchase items the hospital should supply. This is reflected in the quotations:

Sometimes I have to ask a patient's family to buy gloves so I could start an IV. This is discomforting for both me and the patient. —P2

It is appalling that sometimes just simple and basic and essential medications are not available. Just imagine medication paracetamol is stock‐outs. This makes us look incompetent. —P13

#### 5.2.2 Malfunctioning or Obsolete Equipment

Participants recounted working with ageing monitors, suction machines that stalled mid‐procedure, and defibrillators that had been “under maintenance” for years. Reliance on faulty equipment heightened clinical risk and left nurses feeling powerless to guarantee patient safety. This is seen in the quotation

Our monitors are expired and our defibrillators have been ‘under maintenance’ for more than two years; we improvise with CPR and prayer. —P6

And stressed by participant 6

Our suction machine fails mid‐procedure, putting the lives of our patients at risk… you may have to carry oxygen cylinders ward to ward. This makes us worn out. —P9

#### 5.2.3 Personal Out‐of‐Pocket Purchasing of Supplies

In the face of persistent stock‐outs, nurses often used personal funds to buy essential items or turned to private donors forward refurbishments—a practice they labelled “sacrifice” rather than initiative. This is seen in the narrations below

Sometimes I have no option but to buy syringes from my salary because I can't watch mothers bleed. —P1

We have contributed one time to repaint the ward; management called it ‘initiative’ but we call it sacrifice. —P12

### 5.3 Government Neglect and Broken Promises

#### 5.3.1 Unfulfilled Collective Bargaining Agreements

Participants felt betrayed by stalled negotiations over uniform allowances, professional licence renewals, and other contractual benefits. Signed agreements, they said, languished in filing cabinets while frontline staff struggled. This is reflected in the quotations below

There was an agreement and promise concerning uniform allowance adjustment and renewal of our professional pin—but nothing changed. —P5

We have had signed documents sit in drawers while we struggle on the wards for basic necessities. —P10

#### 5.3.2 Communication Gaps and Perceived Disregard

Nurses described repeated meetings with ministry officials that yielded no concrete timelines. Decisions were perceived as top‐down, with grassroots staff learning about policies from radio broadcasts rather than direct briefings. As put it by the participants below

They promise many things that are not implemented… it is just a bunch of meetings that implies nothing. —P14

We only hear about policy decisions on the radio and this lets us feel neglected.

—P17

#### 5.3.3 Politicisation of Health‐Sector Negotiations

Participants believed political cycles, not clinical need, dictated negotiations. Election‐season memoranda quickly evaporated, and divisive tactics were seen as deliberate strategies to weaken collective action. This is seen in the quotation by participants 8 and 19.

During elections they rush to sign MoUs; after elections it's silence. This is nauseating sometimes. —P8

Promises feel like campaign slogans, not contracts… some of the inflammatory statements about forcing nurses to work are disturbing. —P19

### 5.4 Struggle for Professional Recognition

#### 5.4.1 Hierarchical Marginalisation Within the Health Team

Nurses reported entrenched hierarchies in which doctors held managerial power and nursing input was labelled “supportive” rather than clinical, leading to feelings of invisibility and frustration. This is seen in the quotations below.

Doctors are made gatekeepers… nurses are expected to obey without question. This needs a drastic change and an overhaul. —P16

Our ideas are labelled ‘supportive’ rather than ‘clinical’. This makes the public see us as doctors' servants. —P7

#### 5.4.2 Public Misconceptions About Nursing Roles

Participants recounted widespread misunderstanding of nursing as merely pill‐dispensing or physician assistance, reinforcing social devaluation of their expertise.

Most of our patients think we just hand over pills; they don't see the critical decisions we make. —P3

Patients' relatives call me ‘sister’ but treat me like a house help sometimes. —P12

#### 5.4.3 Limited Career Advancement Opportunities

Opportunities for specialty training or promotion were viewed as scarce, politicised, or delayed—particularly in government facilities—fueling resentment and attrition. This is reflected in the quotes

Specialty training slots are scarce and often go to those with connections. —P6

I've been on the same grade for six years despite extra certificates. —P18

### 5.5 Emotional and Psychological Exhaustion

#### 5.5.1 Compassion Fatigue and Burnout

Continuous exposure to suffering, death, and high workloads left many nurses emotionally drained, sometimes too exhausted to engage with their own families after shifts. This is seen in the quotes below

Some days I feel numb, so helpless and with no emotion left to give. —P2

I go home too tired to hug my own children. So, when this is less appreciated, I feel down. —P11

#### 5.5.2 Moral Distress Over Compromised Care

Inability to provide standard‐of‐care treatments due to resource gaps produced feelings of guilt, shame, and professional dissonance. This is seen in the quotations

Watching a patient deteriorate because we lacked oxygen haunts me. —P9

We break our code of ethics daily just to ‘manage’ situations. —P13

#### 5.5.3 Strike as a Form of Self‐Preservation

Striking was framed not merely as industrial action but as a coping mechanism to safeguard mental health and compel authorities to address systemic issues. One of the participants put it

Stopping work is the only way to protect my sanity; striking is the language heeded by authorities. —P4

It is stressed by participant 20

If we don't withdraw our labour, we'll collapse on the job. —P20

The results collectively depict a workforce navigating chronic resource shortages, financial precarity, governmental disengagement, professional marginalisation, and escalating emotional strain conditions that converge to jeopardise both nurse wellbeing and patient care quality.

## 6 Discussion

This study illuminated the complex web of systemic, organisational, and individual‐level challenges confronting nurses in Ghanaian public healthcare facilities. The findings, structured around five thematic categories, reveal how nurses' professional and emotional experiences are shaped by chronic underinvestment, structural neglect, and social misrecognition. Similar to frameworks applied in prior health systems research (Wendel et al. 2015), these results underscore how barriers at multiple levels converge to impact nurses' morale, performance, and retention.

### 6.1 Individual‐Level Impacts

Participants expressed profound emotional and psychological strain arising from the mismatch between their responsibilities and compensation. Nurses reported compassion fatigue and burnout, echoing global trends in high‐stress care environments (Hutsell 2024). Witnessing patient suffering amid inadequate support—such as the inability to administer oxygen or pain relief due to resource scarcity—evoked moral distress, consistent with findings by Gibson (2022), who observed that nurses facing ethically compromising situations often experience guilt and long‐term psychological effects.

Moreover, participants described strikes not as acts of defiance, but as survival mechanisms. These actions were portrayed as the only effective means of communicating urgency to policymakers, resonating with research by Hee et al. (2022), who found that industrial actions among healthcare workers often stem from systemic neglect rather than mere financial dissatisfaction.

### 6.2 Organisational‐Level Constraints

Resource constraints emerged as a major impediment to quality care. Chronic shortages of medications, PPE, and consumables were widely reported. Nurses' accounts of asking patients to procure gloves or buy syringes mirror earlier studies in low‐resource settings, where out‐of‐pocket patient expenses compromise equitable access to care (Melberg 2020).

Participants also lamented malfunctioning or obsolete equipment, leading to unsafe improvisation during emergencies. These infrastructural deficits not only risk patient outcomes but contribute to professional burnout and moral erosion (Owens et al. 2019). The added burden of personal expenditure on hospital supplies—with nurses buying syringes or contributing to ward maintenance—demonstrates a blurring of professional and personal boundaries, reflecting findings by Whiley and Grandy (2022), who highlight the unsustainable emotional labor of healthcare workers in underfunded institutions.

Further, participants highlighted a lack of professional recognition and career mobility. Nurses described hierarchical marginalisation by physicians, aligning with research on health workforce dynamics in sub‐Saharan Africa that point to entrenched medical dominance (Badejo et al. 2020). Misconceptions about nursing roles further devalue nurses' contributions, with the public often viewing them as auxiliary rather than clinical professionals. Limited access to training and promotion compounds this, as career progression is frequently obstructed by favouritism or systemic delays, similar to trends reported by Kuforiji (2024) in other Ghanaian health sector evaluations.

### 6.3 Structural‐Level Deficiencies

At the policy level, nurses perceived a pattern of neglect and broken promises from government actors. Salaries were seen as grossly inadequate, with inflation and currency depreciation further diminishing their value. These economic hardships echo evidence from global health workforce reports that link low and unpredictable pay with poor job satisfaction and attrition (Mohamed et al. 2024).

Delayed or unpaid allowances—such as rural posting and night duty incentives—were interpreted not only as economic strain but as institutional betrayal. Participants repeatedly mentioned how allowances were either politicised or forgotten, leading to a perceived breach of trust. This ties into broader concerns about governance failures and inefficiencies within health sector management in many low‐ and middle‐income countries (Bennett et al. 2018).

Additionally, nurses cited unfulfilled collective bargaining agreements, gaps in communication, and politicisation of negotiations as persistent issues. These findings are corroborated by Christianson et al. (2025), who noted that health worker unions in Ghana often lack genuine engagement from government counterparts. The perception of being “used” during election cycles and forgotten thereafter is a potent example of how institutional trust is eroded in environments where health governance is entangled with political expediency.

## 7 Conclusion

The findings show that nurses' decisions to strike are grounded in lived experiences of institutional neglect, chronic underinvestment, resource scarcity, professional marginalisation, and unfulfilled government commitments. These interconnected conditions shape nurses' motivations for industrial action, which are viewed not as rebellion but as survival strategies in response to untenable working environments. By centring nurses' perspectives, the study illuminates the systemic and institutional factors that precipitate strikes within Ghana's healthcare sector. The findings therefore function as both a diagnostic and advocacy tool, providing evidence to guide policy reforms. Ultimately, the study contributes to improving workforce conditions, strengthening health system resilience, and advancing equitable and sustainable healthcare delivery in Ghana and beyond.

## 8 Limitations

As this study was qualitative in nature, the findings cannot be generalised to the entire nursing population in Ghana. Additionally, the timing of the study during or shortly after industrial actions may have influenced participants' responses, potentially amplifying negative sentiments. The study also did not include views from policymakers, union representatives, or hospital administrators, which could have provided a more balanced perspective. Finally, the scope was limited to urban and peri‐urban areas, with limited institutional diversity.

## Funding

The research had no funding. All costs were borne by the authors.

## Ethics Statement

The research was conducted in accordance with the Declaration of Helsinki. Data collection was done after obtaining approval from the Committee for Human Research Publication and Ethics (*Redacted*) and a written permit from the heads of the selected institution. An informed consent was obtained from all participants.

## Consent

The authors have nothing to report.

## Conflicts of Interest

The authors declare no conflicts of interest.

## Data Availability

The data that support the findings of this study are available from the corresponding author upon reasonable request.
